# A Case Report of 17α-Hydroxylase Deficiency in Two Saudi Siblings With Different Karyotyping

**DOI:** 10.7759/cureus.52191

**Published:** 2024-01-12

**Authors:** Souha Elabd, Ohoud Almohareb, Dania AlJaroudi, Ali Al Zahrani, Imad Brema

**Affiliations:** 1 Obesity, Endocrine, and Metabolism Center, King Fahad Medical City, Riyadh, SAU; 2 Department of Reproductive Endocrine and Infertility Medicine, King Fahad Medical City, Riyadh, SAU; 3 Department of Medicine, King Faisal Specialist Hospital and Research Centre, Riyadh, SAU

**Keywords:** 17α-hydroxylase deficiency, hypokalemia, hypertension, delayed puberty, primary amenorrhea, congenital adrenal hyperplasia

## Abstract

Congenital adrenal hyperplasia (CAH) consists of variable disorders of sex determination and differentiation. 17α-hydroxylase deficiency (17OHD) is an uncommon form of those disorders, which is typically characterized by hypertension, hypokalemia, failure of puberty, and ambiguous genitalia. The 17α-hydroxylase enzyme is encoded by the CYP17A1 gene and it is required for the synthesis of cortisol and sex steroids. The affected females with 17OHD usually present with primary amenorrhea and delayed puberty, which are associated with hypertension and hypokalemia while male patients might show female external genitalia, pseudohermaphroditism, or variable degrees of ambiguous genitalia with intra-abdominal testes in addition to hypertension and hypokalemia as well.

We present two Saudi siblings (19 and 16 years old) who were diagnosed with the rare CAH subtype of 17OHD after presenting with long-standing hypertension, refractory hypokalemia, and failure of puberty. It is interesting that both siblings had biochemical primary adrenal insufficiency; however, both patients did not clinically present with an acute adrenal crisis, which is likely due to the effect of increased levels of deoxycorticosterone. Additionally, although both patients have similar phenotypes and clinical presentations, they have different karyotypes. This again highlights the variability of the manifestations that can result from 17OHD even with an identical mutation in the same family. Both patients were treated successfully with dexamethasone, which has led to the normalization of hypertension, resolution of hypokalemia, and discontinuation of anti-hypertensive medications and potassium supplements after several years of treatment. However, the entire management is quite challenging and requires a multidisciplinary approach regarding difficult issues such as gender identity and assignment and fertility issues in addition to a life-long follow-up.

## Introduction

17α-hydroxylase deficiency (17OHD) is a rare form of congenital adrenal hyperplasia (CAH), which is inherited as an autosomal recessive condition and accounts for less than 1% of all subtypes of CAH [1]. The exact prevalence of 17OHD is not well established; however, it has an estimated incidence of one in 50,000 to 100,000 [2]. To date, more than 500 cases of 17OHD have been reported worldwide [3].

17OHD is caused by a defect in the cytochrome P450 17A1 enzyme, which leads to impaired adrenal and gonadal steroid biosynthesis. This results in compensatory elevation of adrenocorticotropic hormone (ACTH) and overstimulation of the 17-deoxy pathway leading to increased progesterone, corticosterone, and 11-deoxycorticosterone (DOC) synthesis [4]. This enzyme deficiency displays a wide range of clinical manifestations that vary from a full-blown classical type to an asymptomatic form. Misdiagnosis of 17OHD is frequently encountered and can reach more than 90% in some reported series [5]. Therefore, genetic study is crucial for diagnosis. Here, we present two siblings diagnosed to have 17OHD after a prolonged period of hypertension and refractory hypokalemia.

## Case presentation

The index case was a 19-year-old single Saudi female who presented initially to the gynecology clinic in King Fahad Medical City for evaluation of primary amenorrhea, delayed puberty, and absent secondary sexual characteristics. Her past medical history revealed long-standing hypertension since the age of nine years and refractory hypokalemia, for which she was referred to the endocrinology clinic for further evaluation. She was following up in a primary healthcare facility for hypertension and has been treated with amlodipine 5 mg daily. The hypertension was associated with hypokalemia that clinically manifested as intermittent weakness and fatigue and was difficult to manage despite continuous use of oral potassium supplements. She was born after an uneventful pregnancy and had an unremarkable childhood. She is a product of a consanguineous marriage; her parents are second-degree cousins. Family history was remarkable for a younger sister who is 16 years old and under review by a gynecologist for a similar presentation of primary amenorrhea and delayed puberty. Also, she is suffering from hypertension and hypokalemia.

The physical examination of the patient revealed a eunuchoid young lady with a height of 169 cm, weight of 44.5 kg, and body mass index (BMI) of 15.5 kg/m². Blood pressure (BP) was 147/103 mmHg and pulse rate (PR) was 84 bpm. She has generalized skin hyperpigmentation, absent axillary and pubic hair, minimal breast development (Tanner stage 1-2), and normal female external genitalia. The initial laboratory workup for the index case showed hypokalemia in addition to low estradiol levels and elevated gonadotropins, which support the diagnosis of primary gonadal failure (Table 1). Further, an endocrine test revealed low renin and aldosterone levels, excluding the possibility of primary hyperaldosteronism, low cortisol level, high ACTH at around 10 times the normal level, indicating primary adrenal insufficiency, normal 17-hydroxyprogesterone level at 2.8 nmol/L, low androgens, suppressed serum 11-deoxycortisol, and a very high serum DOC, which were consistent with the diagnosis of 17OHD (Table 1).

**Table 1:** Laboratory investigations of the index case, which were done in King Fahad Medical City.

Blood test	Value	Reference range
Serum potassium	2.6	3.5-5.2 mmol/L
Serum sodium	142	135-145 mmol/L
Serum creatinine	41	50-90 mmol/L
Plasma aldosterone	1.8	2.2-35 ng/dl
Plasma renin	<1	1.7-24 ng/L
Aldosterone renin ratio	17.8	<20
Serum cortisol	100	137.9-689 nmol/L
Adrenocorticotropic hormone	113.4	1.6-13.9 pmol/L
Estradiol	70	55.1-1284 pmol/
Luteinizing hormone	44	Prepuberty <1.0 mIU/L
Follicle-stimulating hormone	55	Prepuberty <3.0 mIU/L
Testosterone	0.09	10.4-41.6nmol/L
Dehydroepiandrosterone sulfate	0.11	1.77-9.99 umol/
11-deoxycortisol	<5	<344 ng/dl
17-hydroxyprogesterone	2.83	0-10 nmol/L
Deoxycorticosterone	388	2.0-15 ng/100 ml

**Figure 1:**
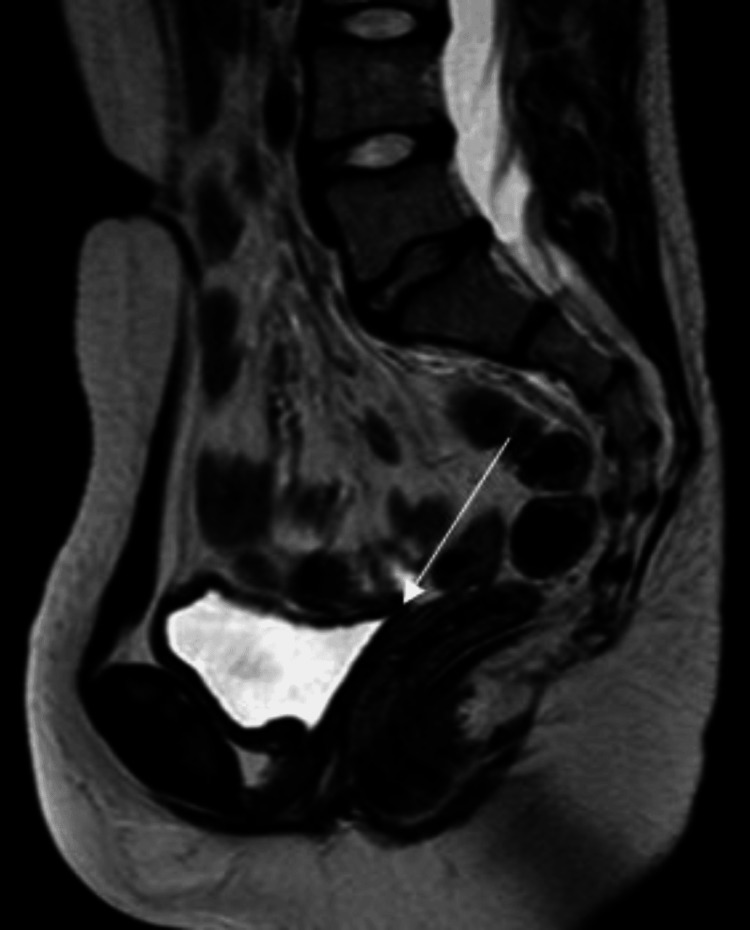
T2-weighted MRI of the pelvis. The sagittal view reveals the absence of the uterus, cervix, and the upper two-thirds of the vagina in the index case (the arrow points to the empty rectovesical pouch).

**Figure 2:**
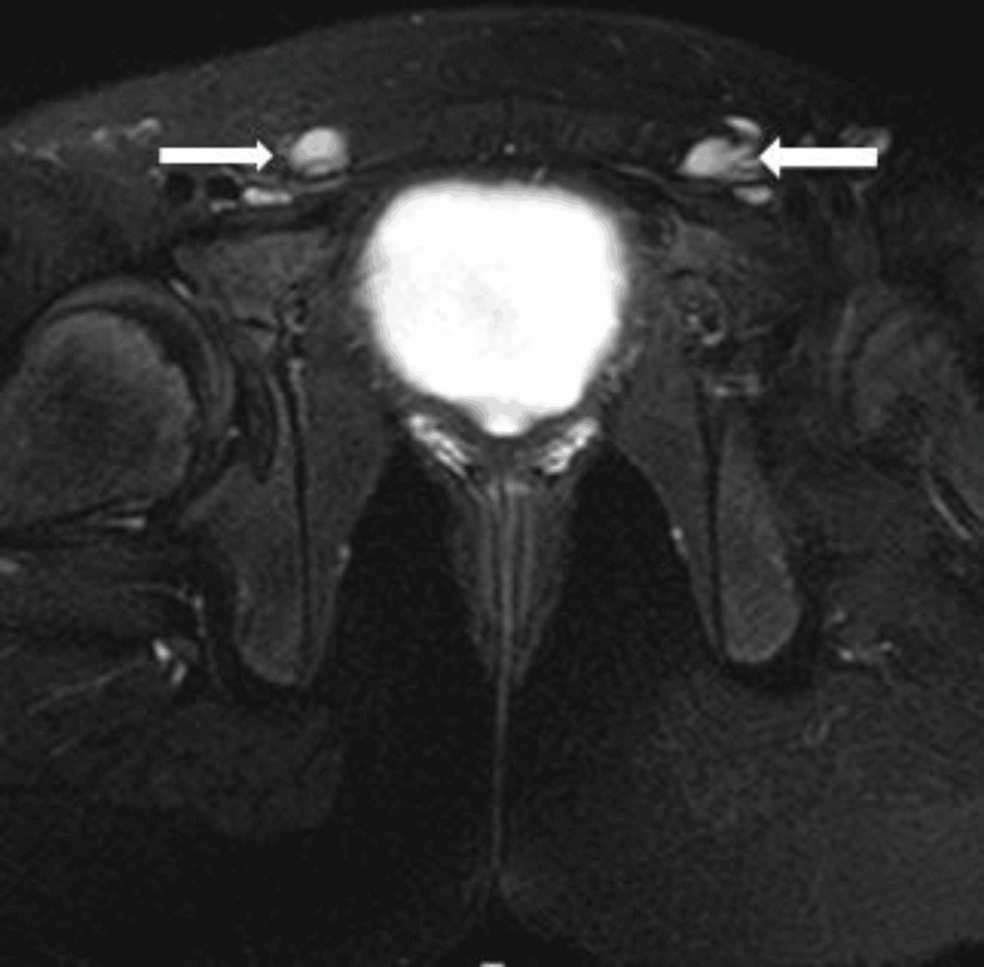
T2-weighted MRI of the pelvis. The axial view shows bilateral testicular tissue in the inguinal canals in the index case (the white arrows).

Pelvic ultrasound examination revealed the absence of female organs, which was confirmed by pelvic magnetic resonance imaging (MRI) that showed complete agenesis and the absence of the ovaries, uterus, cervix, and the upper two-thirds of the vagina (Figure 1). Although the distal third of the vagina was present, it was hypoplastic. Two symmetrical rounded soft tissue lesions were noted within the inguinal canals, each measuring 1.3 cm, representing small undescended testes (Figure 2). The chromosomal analysis confirmed 46,XY male karyotype. Consequently, the genetic study was requested and proved a homozygous nonsense mutation in the CYP17A1 gene (c. 987C>G, p.329 Y>X) confirming the diagnosis of 17OHD in our case.

Management and follow-up

The index case was started on dexamethasone 0.5 mg orally daily, amlodipine and potassium supplements were continued initially, and she was followed up in the endocrine clinic. In her follow-up visit after three months, the BP was normal at 112/67 with a normal potassium level of 3.9 mmol/L off of amlodipine and potassium supplement for the first time, and she felt better with no more fatigue or weakness. However, she gained a significant amount of weight (about 20 kg over one year) and developed mild cushingoid features. Therefore, the dexamethasone dose was decreased to 0.25 mg daily.

The first multidisciplinary team meeting was arranged, including the patient, her family, gynecology, endocrinology, and psychiatry teams. An elaborate and extensive discussion about the diagnosis, the treatment plan, and the possible complications were detailed for the patient and her family. The patient has opted to continue her life as a female, so she was referred to urology and plastic surgery teams and underwent a gonadectomy. Later on, conjugated estrogen 0.625 mg was started, which resulted in the enhancement of the breasts and the development of secondary sexual characteristics.

On the other hand, the 16-year-old younger sister was under evaluation in the gynecology clinic for primary amenorrhea and gonadal dysgenesis. Similarly, she had been on treatment for hypertension and hypokalemia and treated with amlodipine and potassium supplements for a few years. She was referred to the endocrinology clinic for evaluation after confirming the diagnosis of 17OHD in her older sister. The younger sister had a similarly thin body physique with a height of 167 cm, weight of 35 kg, and BMI of 14.3 kg/m² while her blood pressure was 138/78 mmHg. She had no pubic and axillary hair and only minimally developed breasts (Tanner stage 1). The pelvic exam revealed normal female external genitalia, and the vaginal exam suggested dysgenesis.

**Figure 3:**
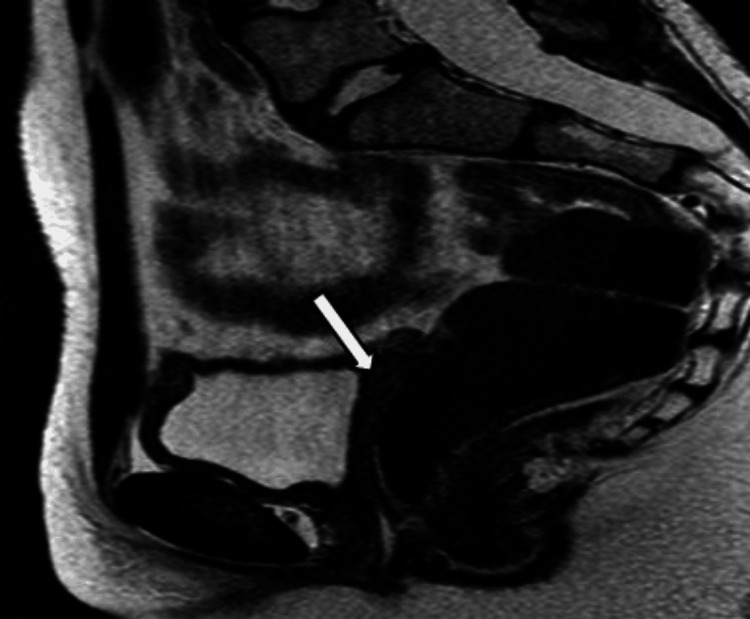
T2-weighted MRI of the pelvis. The sagittal view (in the younger sister) showing the rudimentary uterus (the white-shaded arrow).

The laboratory investigations revealed hypokalemia, low estradiol level, and high gonadotropins along with an elevated ACTH level, extremely low cortisol, low serum androgen levels, and a high serum DOC level, consistent with the diagnosis of 17OHD (Table 2). The pelvic MRI reported the presence of the lower third of the vagina while the upper two-thirds could not be visualized. There was a soft tissue structure at the rectovesical pouch reported as representing a rudimentary uterus (Figure 3). However, there was no visualized ovarian tissue, and the rest of the visualized pelvic structures appear unremarkable.

**Table 2:** Laboratory investigations done in King Fahad Medical City.

Blood test	Value	Reference range
Serum potassium	3.5	3.5-5.2 mmol/L
Serum sodium	137	135-145 mmol/L
Serum creatinine	55	50-90 mmol/L
Plasma aldosterone	20	2.2-35 ng/dl
Plasma renin	<1	1.7-24 ng/L
Aldosterone renin ratio	20	<20
Serum cortisol	9	137.9-689 nmol/L
Adrenocorticotropic hormone	143	1.6-13.9 pmol/L
Estradiol	20	55.1-1284 pmol/L
Luteinizing hormone	41.3	Prepuberty <1.0 mIU/L
Follicle-stimulating hormone	72	Prepuberty <3.0 mIU/L
Testosterone	0.09	10.4-41.6 nmol/L
Dehydroepiandrosterone sulfate	0.11	1.77-9.99 umol/l
11-deoxycortisol	<5	<344 ng/dl
17-hydroxy progesterone	3.61	0-10 nmol/L
Deoxycorticosterone	38	2.0-15 ng/100 ml

The chromosomal analysis confirmed 46,XX female karyotype, and the genetic testing confirmed the presence of the same mutation in the CYP17A1 gene, which was reported in the index case, confirming the diagnosis of 17OHD.

Similarly, the sister was commenced on dexamethasone 0.5 mg daily, which improved her condition and weight (46 kg); however, the dose was decreased later to 0.25 mg. She was followed up regularly in the endocrinology clinic with close observation of her blood pressure and potassium levels, which normalized without the need for anti-hypertensive agents or potassium supplements after a few months. In addition, combined estrogen/progesterone pills were commenced, which led to reasonable development of the breasts and the other secondary sexual characteristics along with cyclical though scanty menstrual periods.

As the disorder is autosomal recessive, which is more prevalent in areas of consanguinity, we have screened the family members, which consisted of the parents and six siblings. The parents are second-degree cousins, and both were carriers of the reported mutation. The youngest sister (10 years old) was found to be a carrier; however, her other siblings refused to be screened. As a result, the family was counseled again about the inherited nature of the diagnosis and the sequences of the disease.

## Discussion

17OHD is a rare autosomal recessive disorder due to mutation in cytochrome P450 17A1 enzyme (CYP17A1), which catalyzes both the 17-hydroxylase reaction, which forms 17-hydroxysteroids, and the 17,20-lyase reaction, which cleaves 21-carbon 17-hydroxysteroids to 19-carbon 17-keto androgen precursors [6]. CYP17A1 is expressed in human adrenals and gonads and such a defect will impair adrenal and gonadal steroid synthesis. The low cortisol levels lead to compensatory high ACTH, which further drives the overproduction of 11-deoxycorticosterone (DOC) and corticosterone. The increased ACTH secretion can induce hyperpigmentation, as in our patient.

The classic presentation of 17OHD includes hypertension and hypokalemia due to high levels of DOC and corticosterone with mineralocorticoid activity in addition to sexual infantilism and pubertal development failure due to the inability to synthesize sex hormones. Males with 17OHD often show pseudohermaphroditism or female external genitalia, and female patients have immature pubertal development and primary amenorrhea [3]. Unlike the classic CAH due to 21-hydroxylase deficiency, patients with 17OHD do not have an adrenal crisis in the postnatal period and symptomatic adrenal insufficiency is rare [7,8] due to mineralocorticoid excess and high corticosterone production. Therefore, the diagnosis is often delayed until hypertension, hypokalemia, or pubertal delay is evaluated during adolescence or early adulthood.

Biochemically, the defect in CYP17A1 causes decreased cortisol levels and subsequently high ACTH that further drives the overproduction of progesterone, DOC, and corticosterone. Therefore, the diagnosis is established by demonstrating elevated DOC (>100 ng/dL (>3 nmol/L)) and corticosterone (>4000 ng/dL (>116 nmol/L)) with low cortisol (<5 mcg/dL (<138 nmol/L)), androgens, and estrogens [7]. Gonadotropins will be elevated secondary to the decline in sex hormones. Patients with 17OHD usually present with low aldosterone levels secondary to high DOC, which suppresses the plasma renin activity [8]. However, aldosterone levels were not low in all cases and some 17OHD cases may have been misdiagnosed as primary aldosteronism (PA) cases [9]. DOC excess also accounts for kaluresis and hypokalemia despite suppressed renin and aldosterone production. Gonadotropins will be elevated secondary to the decline in sex hormones.

It is now well-documented that partial and complete loss of 17-OH activity will result in variable ranges of biochemical and clinical phenotypes [10], and this depends on the type and the site of the mutation. The correlation between the phenotype and genotype is still unknown in patients presenting with 17OHD, and a significant variation in the manifestation and the severity of this disorder were reported even in patients with the same mutation in the CYP17A1 gene [11,12]. Cases of normal or near-normal-looking female genitalia in both males (our index case) and females (our second case) have been well-documented and this has been attributed to variation in the degree of 17OHD and the type of mutations [13,14].

The treatment of 17OHD consists of glucocorticoid replacement and sex steroid hormone supplementation. They are the basic therapy that aims to significantly decrease or normalize the blood levels of ACTH and DOC, which in turn leads to the normalization of the blood pressure and potassium levels [2]. Over three to six months, both siblings have had well-controlled blood pressure off medications along with normal serum potassium levels after several years of problematic, symptomatic hypokalemia that did not optimally respond to potassium supplements. However, close follow-up is important to monitor for the glucocorticoid side effects and dose adjustment. Our patients had significant weight gain after starting dexamethasone for which the dose was reduced to 0.25 mg daily. In general, dexamethasone (0.25-1.0 mg/day), prednisone (2-5 mg/day), or equivalents are recommended [2].

Sex hormone replacement is essential for breast and uterus development and for the maintenance of female secondary sexual characteristics. Estrogen and progestin (for those with an intact uterus) are required in patients with a 46,XX karyotype to induce cyclic withdrawal bleeding and prevent endometrial hyperplasia. In the case of 46,XY patients who decide to continue their lives as males, they should be treated with androgen replacement after consideration of genital reconstructive surgery while those who opted to remain as females should undergo gonadectomy in addition to estrogen replacement to avoid potential malignant transformation in the intra-abdominal testes [3].

Fertility is another challenging issue in female patients with 17OHD, as usually, they cannot conceive spontaneously because of the defect in steroid synthesis, uterus hypoplasia, anovulation due to impaired folliculogenesis, and the lack of mature oocytes [2]. Although some cases of spontaneous menstruation have been described in patients with incomplete 17OHD, very rare cases of fertility and subsequent live births were reported, all of which occurred after the use of assisted reproductive technology and in vitro fertilization (IVF) [15,16]. However, in male patients, the lack of androgen production and testicular atrophy contribute to impaired spermatogenesis and complete infertility [7].

## Conclusions

17OHD is a very rare entity and the diagnosis requires a high index of suspicion due to significant variation in the manifestation and the severity of this disorder even in patients with the same mutation in the CYP17A1 gene, which makes genetic testing an integral part. Management of this condition poses an extra challenging aspect for the physician and the family, taking into consideration the gender assignment and fertility issues of the siblings. Therefore, early multidisciplinary team involvement is very important.
